# Mutations in *KPTN* Cause Macrocephaly, Neurodevelopmental Delay, and Seizures

**DOI:** 10.1016/j.ajhg.2013.10.001

**Published:** 2014-01-02

**Authors:** Emma L. Baple, Reza Maroofian, Barry A. Chioza, Maryam Izadi, Harold E. Cross, Saeed Al-Turki, Katy Barwick, Anna Skrzypiec, Robert Pawlak, Karin Wagner, Roselyn Coblentz, Tala Zainy, Michael A. Patton, Sahar Mansour, Phillip Rich, Britta Qualmann, Matt E. Hurles, Michael M. Kessels, Andrew H. Crosby

**Affiliations:** 1 Monogenic Molecular Genetics, University of Exeter Medical School, St. Luke’s Campus, Magdalen Road, Exeter EX1 2LU, UK; 2 Institute for Biochemistry I, Jena University Hospital and Friedrich Schiller University Jena, D-07743 Jena, Germany; 3 Department of Ophthalmology and Vision Science, University of Arizona School of Medicine, 655 N. Alvernon Way, Tucson, AZ 85711, USA; 4 Wellcome Trust Sanger Institute, Wellcome Trust Genome Campus, Hinxton, Cambridge CB10 1SA, UK; 5 Laboratory of Neuronal Plasticity & Behaviour, University of Exeter Medical School, Hatherly Laboratories, Prince of Wales Road, Exeter EX4 4PS, UK; 6 Windows of Hope Genetic Study, Holmes County, OH 44687, USA; 7 South West Thames Regional Genetics Service, St. George’s Healthcare NHS Trust, London SW17 0QT, UK; 8 Department of Neuroradiology, St. George’s Hospital, London SW17 0QT, UK

## Abstract

The proper development of neuronal circuits during neuromorphogenesis and neuronal-network formation is critically dependent on a coordinated and intricate series of molecular and cellular cues and responses. Although the cortical actin cytoskeleton is known to play a key role in neuromorphogenesis, relatively little is known about the specific molecules important for this process. Using linkage analysis and whole-exome sequencing on samples from families from the Amish community of Ohio, we have demonstrated that mutations in *KPTN*, encoding kaptin, cause a syndrome typified by macrocephaly, neurodevelopmental delay, and seizures. Our immunofluorescence analyses in primary neuronal cell cultures showed that endogenous and GFP-tagged kaptin associates with dynamic actin cytoskeletal structures and that this association is lost upon introduction of the identified mutations. Taken together, our studies have identified kaptin alterations responsible for macrocephaly and neurodevelopmental delay and define kaptin as a molecule crucial for normal human neuromorphogenesis.

## Main Text

Extremes of brain growth have frequently been associated with impaired neurodevelopment and cognition. Occipitofrontal circumference is an indirect measure of brain growth and the one most widely used in clinical practice in which macrocephaly (≥2 SDs above the mean) is indicative of brain overgrowth (megalencephaly) in the absence of hydrocephalus and cranial thickening.^1^ The differential diagnosis of macrocephaly relates to the underlying presence or absence of structural brain anomalies. The strong association between macrocephaly and neurodevelopmental disability, autism, and other pervasive developmental disorders is well recognized.^2–4^ Where macrocephaly is associated with developmental disability, there appears to be a significantly increased risk of seizures.^4^ Over recent years, family studies have begun to identify gene mutations that might cause inherited forms of developmental disability. These studies have shed important new light on the molecular and cellular processes that orchestrate the human neuronal circuitry and that might be dysfunctional in neurological disorders. The establishment of the incredibly intricate human neural circuitry is critically dependent upon a complex and tightly regulated myriad of cellular processes and migrational cues. The actin cytoskeleton is known to play an important role in the formation, propagation, and steering of cell motility and migration during brain development. This in turn leads to the astonishing morphological intricacy that neurons acquire during neuronal differentiation, which is required for the formation of the complex functional neuronal networks underlying human higher brain functions. Mutations in genes encoding molecules important for normal function of the actin cytoskeleton have previously been implicated in inherited forms of developmental disability and brain development,^5–7^ highlighting the important role of the actin cytoskeleton in neuromorphogenesis.

The studies described here derive from the analysis of blood samples obtained for DNA extraction (informed consent was obtained from families from the Anabaptist communities of Ohio according to protocols approved by the institutional review board at the University of Arizona and the Wandsworth Regional Ethics Committee). Nine family members present in four nuclear families were affected by an inherited variable form of neurodevelopmental delay. The most consistent features were global developmental delay, macrocephaly with frontal bossing, high levels of anxiety, and some features suggestive of a pervasive developmental disorder. Additional features included craniosynostosis, recurrent pneumonia, and splenomegally. Neuroimaging was performed in four cases, and no significant intracranial abnormalities were reported. A primary seizure disorder, involving absence or generalized tonic-clonic seizures, was described in three of the nine cases. Dysmorphic features were subtle and included frontal bossing, broad nasal tip, scaphocephaly, hooded eyelids with small, downslanting palpebral fissures, and a prominent chin (Table S1 and Figure S1, available online).

**Figure 1:**
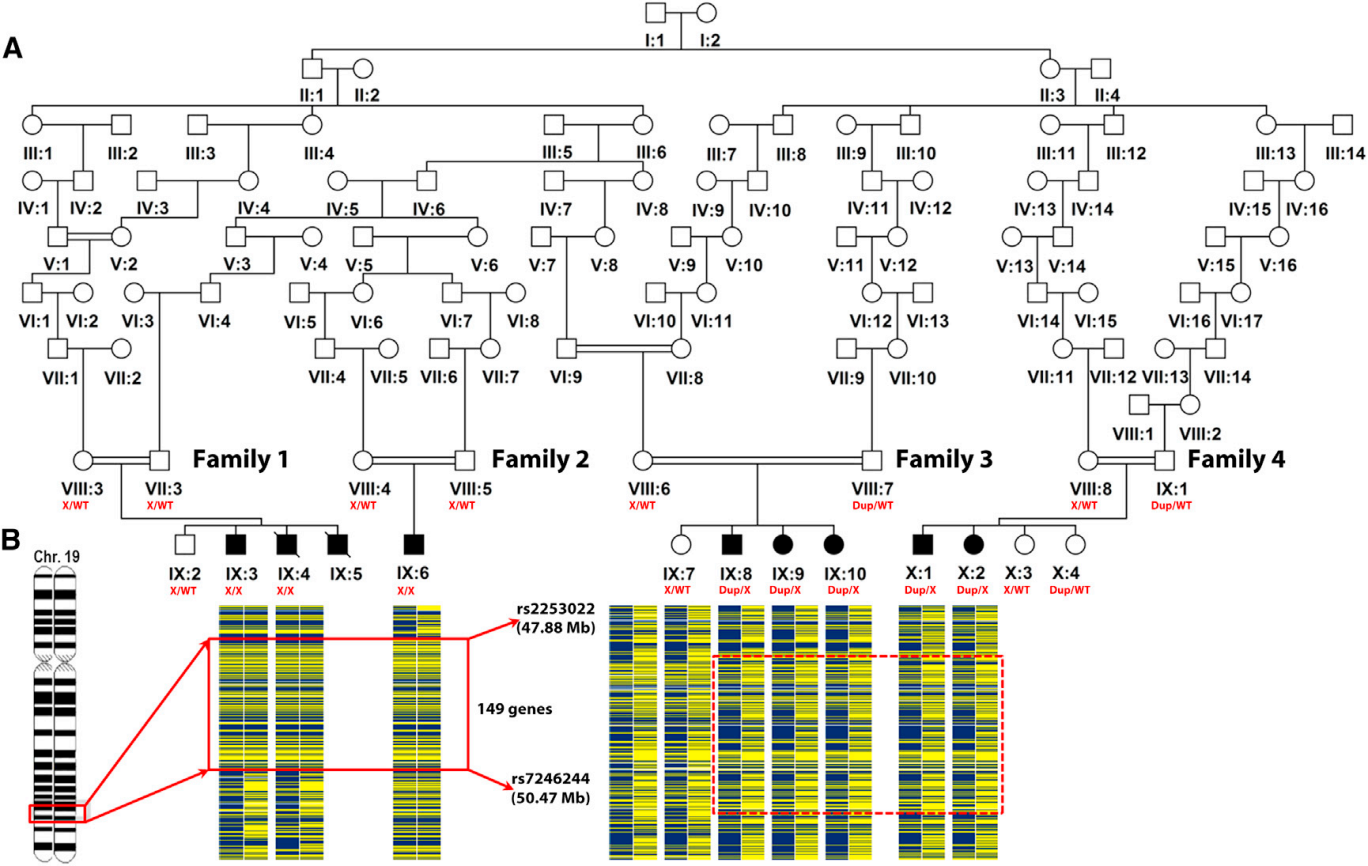
Family Pedigree and Gene Mapping (A) Pedigree diagram showing all four investigated nuclear families (families 1–4), which interlink into a single extended family. Segregation of the two mutations identified (c.776C>A [p.Ser259^∗^] is denoted by X, and c.714_731dup [p.Met241_Gln246dup] is denoted by Dup) is shown (all genotypings were validated by dideoxy sequence analysis). (B) Pictorial representation of the SNP genotype data encompassing the chromosome 19 homozygous (solid box) and compound-heterozygous (dashed box) regions in affected individuals. The locus containing the pathogenic variant is demarcated by SNPs rs2253022 and rs7246244 (2.59 Mb; families 1 and 2).

In order to map the chromosomal location of the pathogenic variant, we genotyped samples from families 1 and 2 genome-wide by using Illumina Human CytoSNP-12 Beadchip arrays incorporating ∼330,000 genetic markers. A single notable homozygous 2.59 Mb region in 19q.13.32 was found to be shared by all affected individuals in families 1 and 2, although no notable homozygous regions were detected in affected members of families 3 or 4 (Figures 1A and 1B). Considered likely to harbor the pathogenic variant in these families, the homozygous region identified in families 1 and 2 is delimited by recombinant SNP markers rs2253022 and rs7246244 and contains 149 genes. Autozygosity across this interval was corroborated by microsatellite-marker analysis in all family members, which defined a haplotype cosegregating with the disease phenotype (data not shown). To identify the causative mutation, we undertook whole-exome sequence analysis of a single affected individual (IX:3, Figure 1A) to generate a profile of variants not present in publically available databases and rare sequence variants. Coding regions were captured with a SureSelect Target Enrichment System (50 Mb) and sequencing on a HiSeq system (Illumina) with 76 bp paired-end reads. We obtained 7.6 Gb of reads, of which 93% had a quality score ≥ 30. Approximately 8% of the reads were marked as duplicates by Picard (v.1.46) and were excluded before mapping to the human genome reference sequence (GRCh37). The Genome Analysis Toolkit (GATK, v.1.0.5777) was used to realign reads near potential indel sites and to recalibrate base qualities; single-nucleotide variants were called with GATK and SAMtools (v.0.1.16), whereas indels were called with GATK and Dindel (v.1.01). All variants were annotated with dbSNP (134) and the 1000 Genomes pilot study (May 2011) for minor allele frequency. The variant consequences on protein structure were predicted by the Variant Effect Predictor (VEP v.2.1) with the use of Ensembl (v.63). Variants were filtered out if the read depth was <4× or >1,200×, if the consensus quality was <20, or if the SNP quality was <25. After filtering, only one likely deleterious variant (g.47983131G>T [NC_000019.9] [c.776C>A (NM_007059.2); p.Ser259^∗^ (NP_008990.2)]) in exon 8 of *KPTN*, encoding the 436 amino acid protein kaptin, was identified within the critical region. The presence of the variant was confirmed by dideoxy sequencing, which also confirmed its cosegregation within families 1 and 2 (Figure 1A). Seven heterozygous carriers were identified in 560 examined control chromosomes, indicating an allele frequency of approximately 0.012 in this community. The variant is also listed in the National Heart, Lung, and Blood Institute (NHLBI) Exome Sequencing Project Exome Variant Server, and one heterozygote has been reported in 8,285 European American chromosomes. We next investigated other Amish families with children showing similar unexplained developmental delay associated with macrocephaly, leading to the detection of the c.776C>A mutation in the heterozygous state in affected members of 2 of 20 such families (families 3 and 4). Compared with this mutation’s occurrence in Amish control studies, this frequency was higher than expected, prompting us to evaluate *KPTN* for a second mutation that could be acting in conjunction with the c.776C>A mutation in these families. Subsequent dideoxy sequence analysis of all coding regions and associated splice junctions of *KPTN* in these families revealed that all affected children were indeed compound heterozygous for the c.776C>A variant, as well as an in-frame 18 bp duplication (g. 47983176_47983193dup [NC_000019.9] [c.714_731dup (NM_007059.2); p.Met241_Gln246dup (NP_008990.2)]) in exon 8. Both the c.776C>A and the c.714_731dup sequence mutations completely cosegregate with the disease phenotype, as would be expected of causative compound-heterozygous mutations (Figure 1A, LOD_MAX_ = 10.33 Simwalk2^8^). The c.714_731dup sequence duplication is not listed in genomic sequence databases, and one heterozygote was detected in 560 Amish control chromosomes.

**Figure 2:**
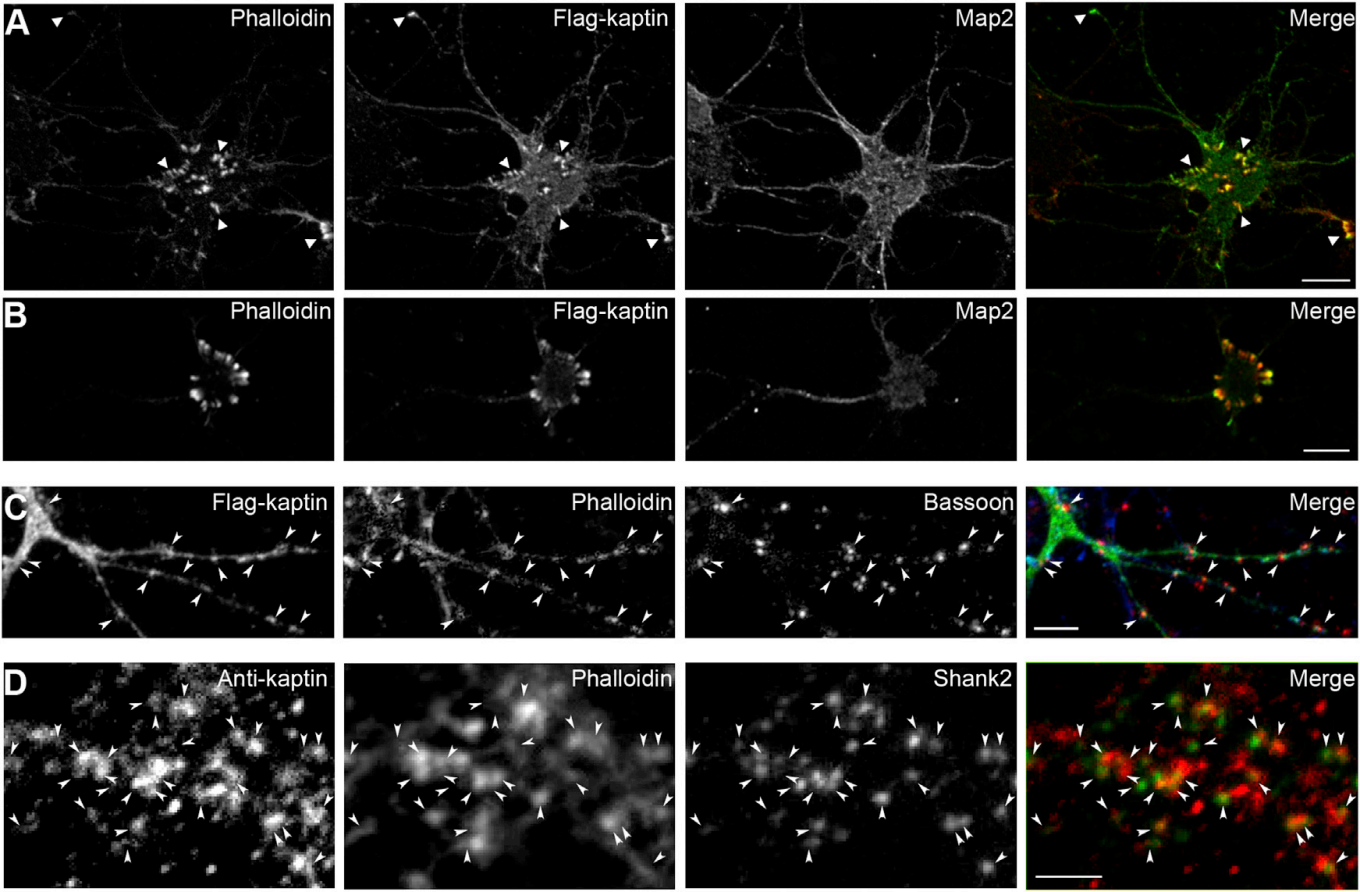
Kaptin Immunolocalization Studies (A and B) Flag-kaptin colocalized with F-actin-rich foci at the cell body and in growth cones (examples of both are marked by arrow heads in A) in DIV5 rat hippocampal neurons transfected at DIV3. For clarity, the anti-MAP2 immunostaining was omitted from the merged images. Scale bars represent 10 μm. (C and D) Endogenous kaptin immunostained together with the presynaptic marker bassoon at DIV14 (C) and with the postsynaptic marker Shank2 at DIV24 (D). Puncta enriched with anti-kaptin immunoreactivity (marked by arrow heads) were rich in F-actin, as shown by fluorescently labeled phalloidin. (C) Furthermore, they were usually contacted by presynapses. The scale bar represents 5 μm. (D) Postsynapses marked by Shank2 were largely positive for both F-actin and anti-kaptin immunolabeling (arrow heads). For clarity, the anti-F-actin staining was omitted from the merged image in (D). High-magnification images are shown. The scale bar represents 2.5 μm.

*KPTN* encodes a largely uncharacterized protein (Figure S2). Our sequence analyses of kaptin identified no protein domains or homologous human proteins that could provide clues to the functional basis of the neurological deficits associated with its alteration. We therefore investigated the expression and localization of kaptin in neurons. In order to do so, we first cloned human kaptin from human embryonic kidney 293 cell cDNA obtained by RNA isolation and RT-PCR as previously described.^9^ Full-length kaptin (amino acids 1–436) was generated by PCR using primers BQ2046 (5′-AAGAATTCATGATGGGGGAGGCG-3′) and BQ2047 (5′-AAGGATCCTTAAGAGGCTGCATT-3′). The PCR product was digested with EcoRI and BamH1 and cloned in-frame into pEGFP-c2 and subcloned into pCMV-Tag2. Primary rat hippocampal cultures were prepared and cultured as previously described.^10,11^ Neurons were transfected with Lipofectamine 2000 (Invitrogen) on days 3, 13, and 23 in vitro, fixed in 4% paraformaldehyde in PBS for 7 min at room temperature 24 and 48 hr after transfection, and processed for immunofluorescence microscopy.^12^ Confocal imaging was performed with a Zeiss Axio Observer equipped with ApoTome and Zeiss Plan-Apochromat 63×/1.4 and 40×/1.3 objectives and an AxioCam MRm CCD camera (Zeiss). Primary rat hippocampal neurons, identifiable by anti-MAP2 immunostaining (Sigma, Abcam), were transfected with wild-type Flag-tagged kaptin (Flag-kaptin) at DIV3 and imaged 2 days later. Wild-type Flag-kaptin was observed to be localized at F-actin-rich foci in close proximity to the cell bodies and at growth cones (Figures 2A and 2B, arrow heads). At later stages of development, when neurons established synapses (DIV14), wild-type Flag-kaptin again colocalized with F-actin-rich sites. Along dendrites, these sites appeared mainly to represent F-actin-rich postsynapses, given that almost all kaptin-enriched puncta were contacted by presynaptic structures containing bassoon (a marker for presynaptic active zones) (Figure 2C). To be able to undertake immunolabeling experiments of endogenous kaptin, we first characterized polyclonal rabbit anti-kaptin (Sigma) in COS-7 cells. Cells expressing wild-type Flag-kaptin were highlighted by anti-kaptin immunolabeling (Figure S3A). Coimmunostaining of primary rat hippocampal neurons at DIV24 with anti-kaptin and anti-Shank2 (Neuromab) demonstrated that the dendritic accumulations of Flag-kaptin at F-actin-rich puncta are indeed of physiological relevance and reflect the localization of endogenous kaptin at postsynapses. The vast majority of anti-kaptin-immunolabeled puncta were not only enriched with F-actin but were additionally immunopositive for Shank2, a postsynaptic scaffold protein interacting with F-actin binding proteins;^12,13^ Figure 2D). Kaptin thus appears to be associated with dynamic actin cytoskeletal structures of neuronal cells. Consistent with this, wild-type Flag-kaptin accumulated at COS-7 cell lamellipodia (Figure S3B), the subcellular regions marked by dense arrays of dynamic actin filaments in mobile fibroblasts. In neurons, the cortical actin cytoskeleton is known to be important for proper neuronal-network formation during development. Our observations therefore suggest a role for kaptin in neuromorphogenesis.

**Figure 3:**
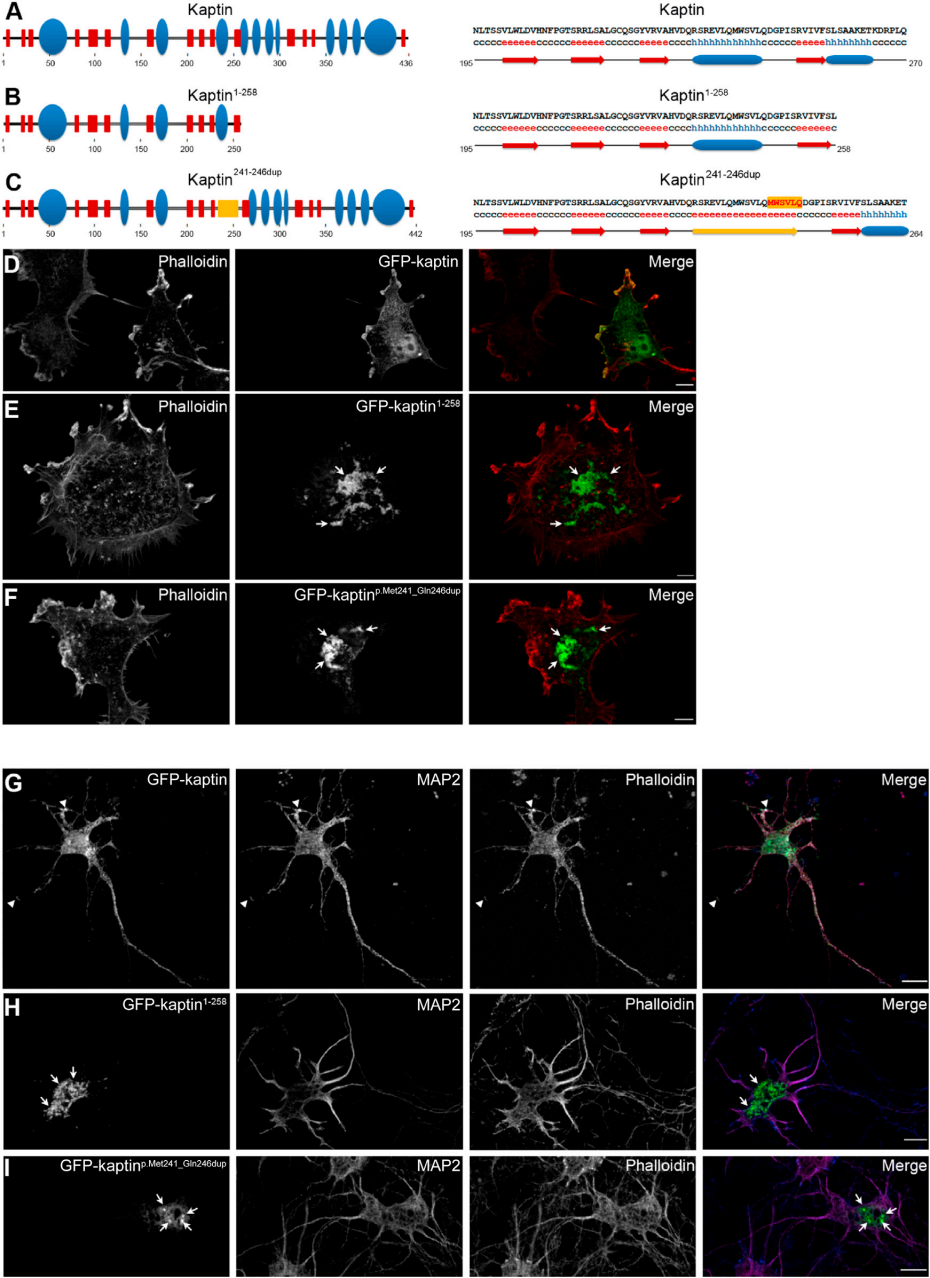
Immunolocalization Studies of Altered Kaptin (A–C) Schematic representation of wild-type human kaptin (A), the truncated p.Ser259^∗^ (c.776C>A; GFP-kaptin^1–258^) (B), and the duplication (GFP-kaptin^p.Met241_Gln246dup^) (C). On the left is a graphic overview of secondary structures; β sheets are shown as red boxes, and α-helices are shown as blue ellipsoids. On the right are amino acids and secondary-structure elements around amino acid 230. α-helix 4 is predicted to be converted into a β sheet by the insertion of amino acids 241–246, as shown in yellow. (D–F) Localizations of GFP-kaptin, GFP-kaptin^1–258^, and GFP-kaptin^p.Met241_Gln246dup^ (green in merges) in COS-7 cells counterstained with phalloidin (red in merged images). Note that whereas wild-type kaptin was distributed in the cytosol and accumulated at F-actin-rich lamellipodia (D), both alterations showed accumulations at perinuclear regions (E and F). (G–I) Transfection of DIV3 rat hippocampal neurons with wild-type and altered kaptin (cells were fixed and imaged at DIV5). Wild-type kaptin was found throughout the cell and showed accumulations at growth cones and F-actin-rich sites of the cell body (G; arrow heads). In contrast, both alterations accumulated in areas of the cell body (H and I; arrows). Merged images show wild-type and altered kaptin in green, MAP2 in red, and phalloidin in blue. Scale bars represent 10 μm.

The c.776C>A sequence variant is predicted to introduce a premature stop codon and result in loss of function as a result of degradation of the mutated transcript by mRNA-surveillance mechanisms; however, because of a lack of patient material, we have been unable to confirm whether a truncated protein (lacking the C-terminal amino acids 259–436) is produced. In contrast, the in-frame c.714_731dup mutation is likely to result in the insertion of six amino acids (Met-Trp-Ser-Val-Leu-Gln) into the full-length kaptin. In order to investigate the functional outcome of this mutation, we undertook in silico analysis of the secondary structural elements of wild-type and altered kaptin. This revealed that the N-terminal half of kaptin is likely to comprise a series of relatively densely organized β sheets, interspersed by only three α helices, and becomes more α-helical starting with α-helix 4 (spanning amino acids 234–245; Figure 3A). Sequence and predicted structural conservation of both the N-terminal and C-terminal portions of kaptin are very high even between evolutionarily distant mammalian species (Figure S2). Strikingly, duplication of the six amino acids (241–246) is predicted to disrupt α-helix 4 and result in its conversion into an extended β sheet (Figure 3C) and is therefore likely to have a profound effect on kaptin function.

In order to experimentally explore the functionality of any protein arising from translated mutant transcripts, we generated both disease-associated GFP-tagged mutants. We generated the p.Met241_Gln246dup altered kaptin (GFP-kaptin^p.Met241_Gln246dup^) by fusing an N-terminal portion carrying the duplication and a SmaI site introduced as a silent mutation (primers BQ2046 [5′-AAGAATTCATGATGGGGGAGGCG-3′] and BQ2050 [5′-TCACCCGGGAGATGGGCCCGTCTTGCAACACGCTCCACATCTGCAGGACCGACCAC-3′]) with a C-terminal portion containing a SmaI site also introduced by silent mutation (primers BQ2048 [5′-ATCTCCCGGGTGATTGTGTTCAG-3′] and BQ2047 [5′-AAGGATCCTTAAGAGGCTGCATT-3′]). We generated the p.Ser259^∗^ altered kaptin (GFP-kaptin^1–258^) by PCR using primers BQ2046 (5′-AAGAATTCATGATGGGGGAGGCG-3′) and BQ2070 (5′-AAGTCGACCtAGAGGCTGAACAC-3′). PCR products were cloned in-frame into pEGFP-c2. Whereas wild-type GFP-tagged kaptin (GFP-kaptin) was found to localize at F-actin-rich lamellipodia of COS-7 cells, both altered forms of kaptin displayed no F-actin association but instead accumulated at irregular perinuclear sites (Figures 3D–3F). GFP-kaptin^1–258^ showed a more pronounced tendency to form such accumulations; almost all cells were marked with little additional cytoplasmic staining outside of these foci. Alternatively, GFP-kaptin^p.Met241_Gln246dup^ typically displayed fewer and slightly smaller accumulations; there were slightly higher levels of cytoplasmatic staining outside of these foci (Figures 3D–3F). This indicates that both proteins are likely to be nonfunctional, although we cannot exclude the possibility that misfolded altered protein might accumulate in neurons of affected individuals and lead to dominant-negative effects on other neuronal proteins or cell processes.

Finally, because *KPTN* mutations result primarily in a form of neurodevelopmental disease, we also analyzed the behavior of the altered forms of kaptin in primary hippocampal neurons during early development. Whereas wild-type GFP-kaptin was again found to colocalize with dynamic F-actin in growth cones and foci at the cell body, both GFP-kaptin^1–258^ and GFP-kaptin^p.Met241_Gln246dup^ were found to accumulate in a manner reminiscent of the COS-7 studies in the cell body or at perinuclear sites (Figures 3G–3I).

We investigated a number of families from the Anabaptist communities of Ohio and found that multiple individuals aged 1–30 years were affected by a syndrome in which the cardinal features include macrocephaly, global developmental delay, behavioral abnormalities, and seizures. Our molecular studies determined that two distinct founder mutations affecting the same gene (*KPTN*, encoding kaptin), both of which have become entrapped within the community, are responsible. Compared with individuals found to be compound heterozygous for p.Ser259^∗^ and p.M241_Q246dup, those individuals found to be homozygous for the p.Ser259^∗^ nonsense alteration appeared to be more severely affected given that they had a higher incidence of seizures and a greater degree of intellectual impairment. This might indicate that p.Met241_Gln246dup retains a limited functionality in vivo, and perhaps consistent with this, we observed that p.Met241_Gln246dup showed a slightly lesser tendency than p.Ser259^∗^ to form perinuclear accumulations in our transfection studies. However, the sample cohort is currently too small for confidently determining any genotype-phenotype correlation.

Kaptin is a largely uncharacterized protein originally isolated from human blood platelets but subsequently found to be expressed in fibroblasts and intestinal and sensory epithelia.^14^ A previous study of this molecule suggested a role at stereocilia tips, and so *KPTN* was proposed as a candidate gene for hearing loss.^15^ However, the affected individuals described in this study have no evidence of sensorineural hearing deficits. During development, the actin cytoskeleton plays a pivotal role in neuronal cell morphology and migration, including the generation, protrusion, and steering of growth cones and the formation of postsynapse and dendritic spines.^16–18^ Our studies confirm kaptin expression in neuronal (MAP2-positive) cells. Given that kaptin was found to localize to F-actin-rich structures, it is conceivable that loss of kaptin function could either directly or indirectly lead to impairment of the neuronal actin cytoskeleton, required for dendritic arborization and/or spine formation, and result in the disease phenotype described. Support for this has been provided by studies of Rab39B, a small GTPase associated with the Golgi apparatus;^19^ alterations in this protein lead to its downregulation and a concomitant reduction in dendritic arborization and synapse formation. This was previously associated with a disease phenotype comprising mental retardation, epilepsy, and macrocephaly,^20,21^ features which overlap with those described here as arising from kaptin alterations. Similarly, deficiencies of Rho GTPases, which regulate the actin cytoskeleton by a growing variety of effector proteins, have been associated with intellectual disability and defects in spine structure.^22–25^ Several other actin-associated proteins, including drebrin A, cortactin, and Abp1, have also been found to decrease spine density or formation,^26–28^ and a growing body of evidence supports a role for the Arp2/3 complex and directly and indirectly associated proteins in postsynapse formation and proper development of neuronal morphology.^10,11,27,29–38^

Our analyses reveal that wild-type kaptin is enriched in neuronal growth cones and at discrete cortical sites of neurons at early developmental stages. Furthermore, wild-type kaptin was found to accumulate at postsynapses of neurons undergoing synaptogenesis (DIV14), as indicated by spatial correlation along dendrites of kaptin accumulations by phalloidin and synaptic-marker immunostaining. Consistent with this, kaptin was also found to be present in the postsynapses of mature neurons. The sites demarcated by kaptin localization represent areas of high F-actin content and high actin dynamics. An association between kaptin and dynamic F-actin was also indicated by the observed accumulations of Flag-kaptin at the dynamic lamellipodia as opposed to the more static stress fibers in COS-7 cells. These observations are consistent with the suggestion of a lamellipodial localization of kaptin in chicken embryonic fibroblasts and with the original isolation of kaptin from blood cells with the use of F-actin columns.^14^

Taken together, our studies indicate that both of the identified *KPTN* mutations are likely to result in loss of function of kaptin, either by degradation of the mutant transcript via mRNA-surveillance mechanisms (c.776C>A) or by the production of mislocalized and/or nonfunctional protein products. These *KPTN* mutations result in a distinctive clinical syndrome, and the presence of macrocephaly combined with global developmental delay should prompt the diagnostic analysis of *KPTN* in affected individuals from Anabaptist communities. The potential benefits of early diagnosis in this condition are indicated by the improvement in developmental markers in our study’s youngest two affected individuals, both of whom received early and intensive developmental interventions, although the lack of seizures in these individuals might also have been beneficial. Finally, our identification of two *KPTN* founder mutations within this Anabaptist population parallels the situation seen for a number of other genes with multiple mutations that also commonly cause inherited diseases globally (e.g., *GJB2* mutations in inherited hearing loss, and *ATM* mutations in ataxia telangiectasia), indicating that kaptin developmental delay might be similarly widespread.

## Web Resources

The URLs for data presented herein are as follows:

1000 Genomes, http://browser.1000genomes.org/index.html
Ensembl Genome Browser, http://www.ensembl.org/index.html
GATK, http://www.broadinstitute.org/gatk/about/citing-gatk
NCBI, http://www.ncbi.nlm.nih.gov/
NHLBI Exome Sequencing Project (ESP) Exome Variant Server, http://evs.gs.washington.edu/EVS/
Online Mendelian Inheritance in Man (OMIM), http://www.omim.org
PolyPhen-2, http://genetics.bwh.harvard.edu/pph2/
PROVEAN, http://provean.jcvi.org/seq_submit.php
PubMed, http://www.ncbi.nlm.nih.gov/pubmed/
SAMtools, http://samtools.sourceforge.net
SIFT, http://sift.jcvi.org/
Variant Effect Predictor (VEP), http://useast.ensembl.org/info/docs/tools/vep/index.html
UCSC Human Genome Browser, http://www.genome.ucsc.edu
