# Supplementary material for: Mutations in KPTN Cause Macrocephaly, Neurodevelopmental Delay, and Seizures
Source: Am J Hum Genet. 2014 Jan 2;94(1):87–94. doi: 10.1016/j.ajhg.2013.10.001 (PMC3882725; doi:10.1016/j.ajhg.2013.10.001)
Supplement: Document S1. Figures S1–S4 and Table S1 [file mmc1.pdf]

The American Journal of Human Genetics, Volume *94*

Supplemental Data

Mutations in *KPTN* Cause Macrocephaly,

Neurodevelopmental Delay, and Seizures

Emma L. Baple, Reza Maroofian, Barry A. Chioza, Maryam Izadi, Harold Cross, Saeed Al-Turki, Katy Barwick, Anna Skrzypiec, Robert Pawlak, Karin Wagner, Roselyn Coblentz, Tala Zainy, Michael A. Patton, Sahar Mansour, Phillip Rich, Britta Qualmann, Matt Hurles, Michael M. Kessels, and Andrew H. Crosby

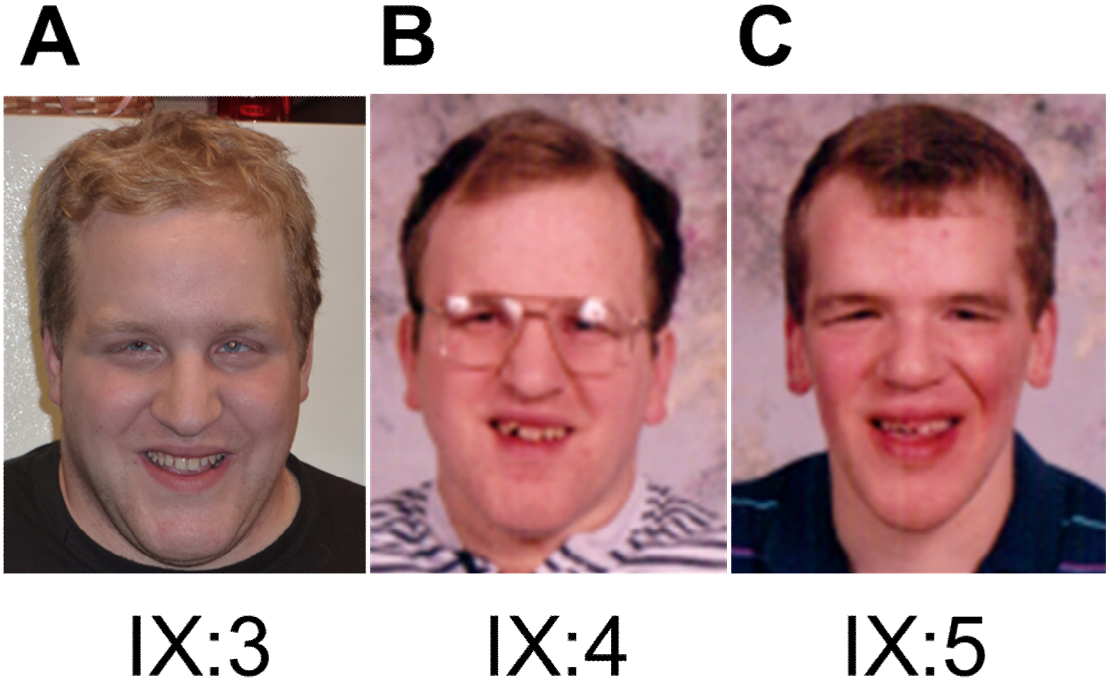

Figure S1.
Panels **A-C** show the facial features of individuals homozygous for *KPTN* p.Ser259*, which include macrocephaly, frontal bossing with a high frontal hairline, long face with a prominent chin, down-slanting, small palpebral fissures, broad nasal tip, and hooded eye lids.
Published with consent.

```
Human           MMGEAAVAAGPCPLREDSFTRFSSQSNVYGLAGGAGGRGELLAATLKGKVLGFRYQDLRQ 60
Armadillo       -MGEAAVAAGPCPLREDSFTRFSSQSNVYGLAGGAGGRGELLAATLKGKVLGFRYQDLRQ 59
Dolphin         -MGEAAVAAGPCPLREDSFTRFSSQSNVYGLAGGAGGRGELLAATLKGKVLGFRYQDLRQ 59
Mouse           -MGEAAVAEGPCPLLEDSFTRFSSQSNVYGLAGGADGRGELLAATLKGKVLGFRYQDLRQ 59
                 ******* ***** ********************.************************

Human           KIRPVAKELQFNYIPVDAEIVSIDTFNKSPPKRGLVVGITFIKDSGDKGSPFLNIYCDYE 120
Armadillo       KIRPVAKELQFNYIPVDAEIVSIDTFNKSPPKRGLVVGITFIKDSGDKGSPFLNIYCDYE 119
Dolphin         KIRPVAKELQFNYIPVDAEIVSIDTFNKSPPKQGLVVGITFIKDSGDKGSPFLNIYCDYE 119
Mouse           KIRPVAKELQFNYIPVDAEIVSIDTFNKSPPKRGLVVGITFIKDSGDKGSPFLNIYCDYE 119
                ********************************:***************************

Human           PGSEYNLDSIAQSCLNLELQFTPFQLCHAEVQVGDQLETVFLLSGNDPAIHLYKENEGLH 180
Armadillo       PGSEYNLDSIAQSCLNLELQFTPFQLCHAEVQVGDQLETVFLLSGNNPAIHLYKENEGLH 179
Dolphin         PGSEYNLDSIAQSCLNLELQFTPFQLCHAEVQVGDQLETVFLLSGNDPAIHLYKENEGLH 179
Mouse           PGSEYNLDSIAESCLNLELQFTPFQLCHAEVQVGDQLETVFLLSGNDPAIHLYKENEGLH 179
                ***********:*********************************:**************

Human           QFEEQPVENLFPELTNLTSSVLWLDVHNFPGTSRRLSALGCQSGYVRVAHVDQRSREVLQ 240
Armadillo       QFEEQPVENLFPELTHLTSSVLWLHVHNLPGTSRRLSALGCQSGYVRVAHVDQRSREVLQ 239
Dolphin         QFEEQPVENLFPELTNLTSSVLWLDVHNLPGTSRRLSALGCQSGYVRVAHVDQQSREVLQ 239
Mouse           QFEEQPVENLFPELTNLTSSVLWLDVHNLPGSSQRLSALGCQSGYVRVAHVDQKNQEILQ 239
                ***************:********.***:**:*:*******************:.:*:**

Human           MWSVLQDGPISRVIVFSLSAAKETKDRPLQDEYSVLVASMLEPAVVYRDLLNRGLEDQLL 300
Armadillo       TWTVLQDAPISRVTVFNLAAPEESTERPQQEEYSVLVASMLEPAVVYRDLLRRGLEDQLL 299
Dolphin         TWTILQDGPISRVIVFSLSAPEETEDRPQREEYSVLVASMLEPAVVYRDLLSRGLEDQLL 299
Mouse           TWTIQQDGPISRVIVFSLSASEATQDSPQQEGYSLLVASMLEPAVVYWDLLNKGLDDQLL 299
                 *:: **.***** **.*:*.: : : * :: **:************ *** :**:****

Human           LPGSDQFDSVLCSLVTDVDLDGRPEVLVATYGQELLCYKYRGPESGLPEAQHGFHLLWQR 360
Armadillo       LPGSDQFDSVLCGLVTDVDLDGRPEVLVATYGQELLCYKYCA-------AGQGFRLLWRR 352
Dolphin         LPGSDQFDSVLCGLVTDIDLDGRPEVLVATYGQELLCYKYCSPGRGLPGAQRGFRLLWQR 359
Mouse           LPGSDQFDSVLCGLVTDVDLDGQPEVLVATYGQELLCYKYRG----LPEDSRGFRLLWRR 355
                ************.****:****:***************** .         :**:***:*

Human           SFSSPLLAMAHVDLTGDGLQELAVVSLKGVHILQHSLIQASELVLTRLRHQVEQRRRRLQ 420
Armadillo       SFSSPLLAMADVDLTGDGLRELAVVSLKGMHILQHSLVQASELALTRLRRQVEQRRRRPP 412
Dolphin         SFSSPLLAMAHVDLTGDGLRELAVVSLKGVHILQHSLIQASELVLTRLRHQVEQRRRQPQ 419
Mouse           SFASPLLAMAHVDLTGDGLRELAVISLKGVHILQHSLIQASELVLTRLRHQVEQRK-HQQ 414
                **:*******.********:****:****:*******:*****.*****:*****: :

Human           GLEDGAGAGPAENAAS 436
Armadillo       GLGARAGPGAAENPAS 428
Dolphin         RLGDRVGAGPAETPAS 435
Mouse           GLGDRVGPRPVEHPAS 430
                 *   .*. ..* .**
```

Figure S2.
Alignment of kaptin proteins from diverse mammalian species including human (gi|108936022|sp|Q9Y664.2),armadillo(gi|488582075|ref|XP_004476450),dolphin(gi|470599504|ref|XP_004311953) and mouse(gi|148710164|gb|EDL42110.1), revealing an almost complete sequence conservation of the N-terminal half of kaptin and an also very high conservation of the C-terminal half. Residues corresponding to kaptin amino acids 1-258 are shaded in grey, residues duplicated in the p.Met241_Gln246dup mutant are highlighted in yellow.

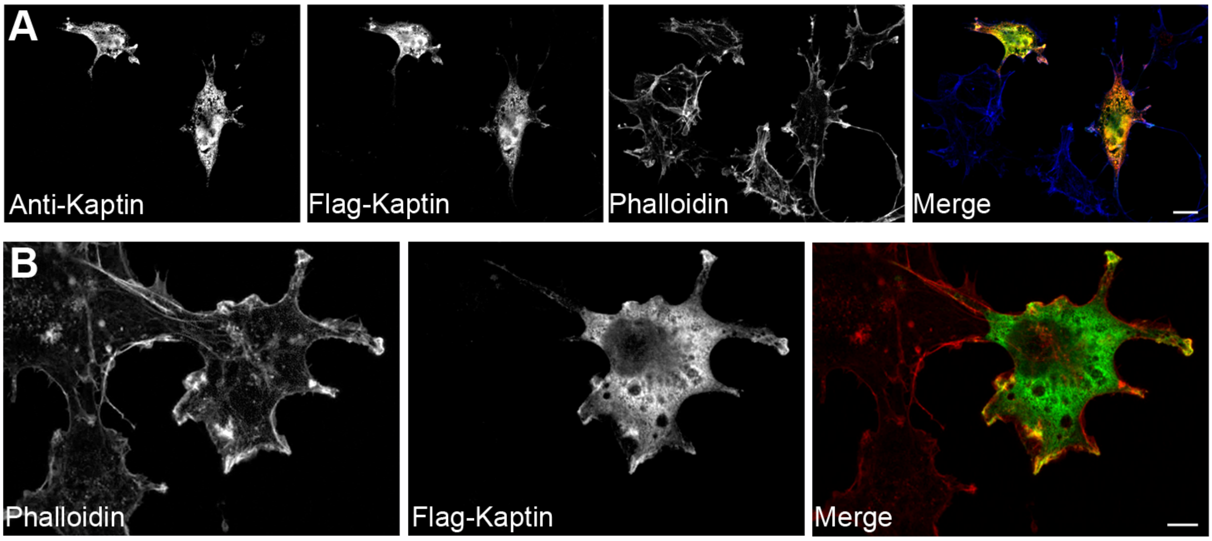

Figure S3.

A Flag-kaptin (green in merge) in COS-7 cells is enriched in F-actin-rich lamellipodia (red in merge) and additionally shows a cytoplasmic localization.

B, Anti-kaptin antibody characterization using Flag-kaptin overexpression in COS-7 cells. Note that transfected cells were recognized by anti-kaptin antibodies, that untransfected cells showed no significant immunolabeling and that there was a good spatial overlap of Flag-kaptin and anti-kaptin detection. Bars, 10 µm.

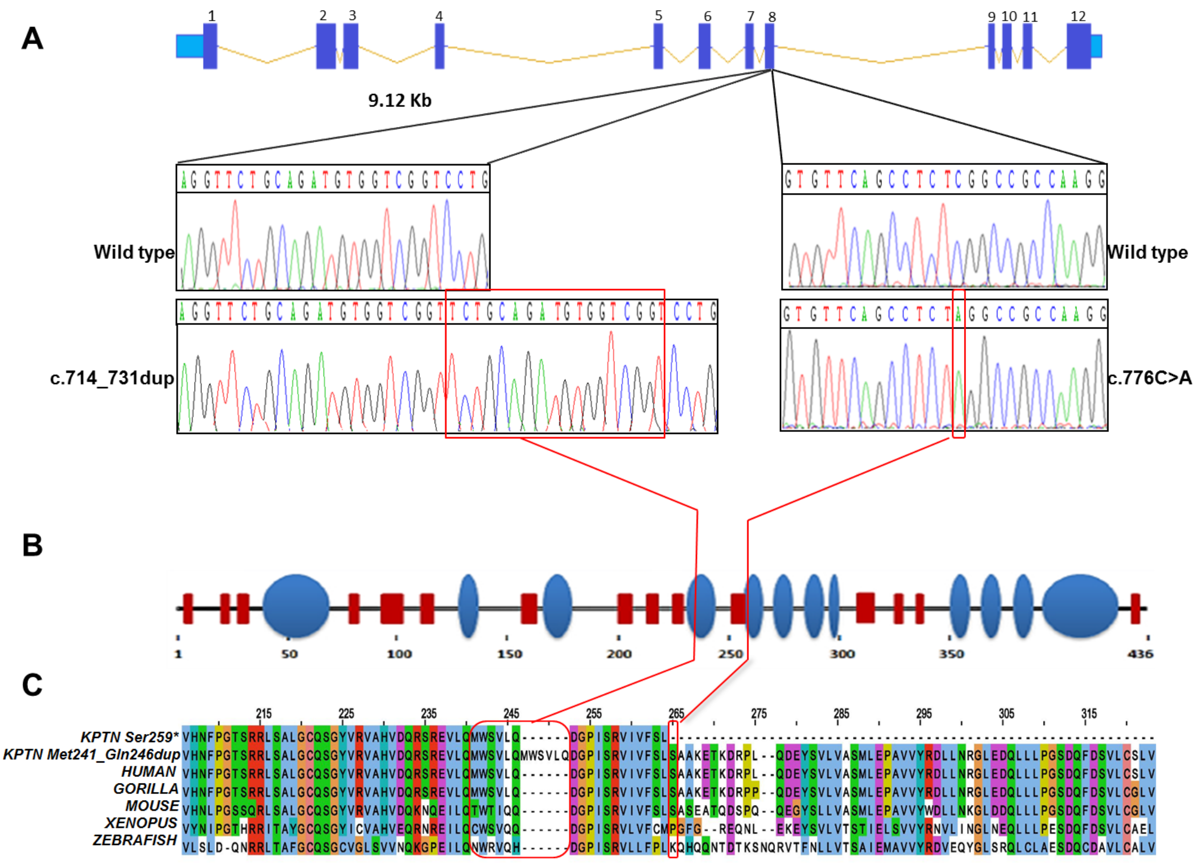

Figure S4.
A; Genomic organization of the *KPTN* and position of the two exon 8 *KPTN* mutations (c.776C>A; c.714-731dup) identified and translated outcomes (p.Ser259*; p.Met241_Gln246dup) in the corresponding polypeptide products (**B**, **C**). Red boxes denote beta-sheets and blue elipoids denote alpha-helices.

Table S1. A comparison of clinical findings of affected individuals homozygous or compound heterozygous for *KPTN* mutations

	IX:3	IX:4	IX:5	IX:6	IX:8	IX:9	IX:10	X:1	X:2
**Genotype**	p.Ser259*/p.Ser259*	p.Ser259*/p.Ser259*	p.Ser259*/ p.Ser259*	p.Ser259*/ p.Ser259*	p.Ser259*/ p.Met241_Gln246dup	p.Ser259*/ p.Met241_Gln246dup	p.Ser259*/ p.Met241_Gln246dup	p.Ser259*/ p.Met241_Gln246 dup	p.Ser259*/ p.Met241_Gln246dup
**Gender**	M	M	M	M	M	F	F	M	F
**Age at evaluation (years)**	28.2	Deceased age 29 yrs (head injury)	Deceased age 30 yrs (pneumonia)	16.5	13.2	22.7	24.9	1.4	7.8
**Growth parameters**									
Birth weight kg (SDS)	2.95(0.7)	2.92 (0)	3.46 (1.3)	1.59 (-0.2)	3.35(-0.4)	2.89 (-1.2)	3.16 (-0.5)	3.2 (2.0)	2.75 (1.1)
Birth OFC cm (SDS)		40.6 at 6 wks (2.9)	35.6 (1.75)		51 at 10mo (3.9)			"macrocephaly"	"macrocephaly"
Height cm (SDS)	166.7cm (-1.6)	N/K	165.1 (-1.8) at 19.1yrs	169 (-0.7)	161.6 (0.6)	156.2 (-1.3)	160 (-0.6)	NK	123.5 (-0.4)
Weight kg (SDS)	121.2 (3.4)	N/K	66.5 (-0.2) at 19.1yrs	63.1 (0.1)	51.5 (0.8)	107.9 (3.6)	82 (2.2)	NK	23.1 (-0.5)
OFC cm (SDS)	62 (3.0)		63.5 (3.6) at 19.1yrs	62.5 (3.4)	61 (3.3)	63 (5.4)	60 (3.2)	52.5 (3.0)	55.4 (2.1)
Parental OFC cm (SDS)		Mother 55.5 (0.1) Father 60 (1.7)		Mother 58 (1.9) Father 59 (1.2)		Mother 58.5 (2.3)		Mother 57 (1.2) Father 59.5 (1.5)	
**Development**									
Walked (years)	1	1	1.33	1.9	4	3.8	2.4	>2.2	2.2
Expressive & receptive language deficit	✓	✓	✓	✓	✓	✓	✓	✓	✓
Intellectual disability	Moderate	Mild/Moderate	Severe	Moderate	Moderate	Moderate IQ 45	Moderate IQ 42	Mild	Mild
**Neurology**									
Childhood hypotonia	-	-	-	✓	✓	✓	✓	✓	✓
Seizures	onset age 3 mo AS and GTCS	onset age 7 yrs AS and GTCS	onset age 7 yrs GTCS	-	-	-	-	-	-
Neuroimaging	MRI - normal[a]	N/A	CT mild ventriculomegaly[a]	N/P	N/A	N/P	N/P	CT– widening of the metopic suture	CT normal
Behavioural characteristics	Repetitive speech Anxiety		Stereotypies	Stereotypies Hyperactivity Anxiety	Stereotypies Repetitive speech Anxiety	Stereotypies Repetitive speech Anxiety	Stereotypies Repetitive speech Anxiety	Phobias Anxiety	Anxiety
**Physical anomalies**									
Head shape	Frontal bossing Prominent chin	Frontal bossing Prominent chin	Frontal bossing Prominent chin	Frontal bossing Scaphocephaly Prominent chin Hypertelorism	Scaphocephaly Sagittal synostosis- operated age 10mo Frontal bossing	Frontal bossing	Scaphocephaly Frontal bossing	Plagiocephaly Frontal Bossing Hypertelorism	Prominent chin Frontal bossing
Other physical findings	5th finger clinodactyly	5th finger clinodactyly	5th finger clinodactyly				Hepatosplenomegally Liver cirrhosis recurrent pneumonia in childhood	Splenomegally Anaemia Fetal finger pads recurrent pneumonia	Fetal finger pads

Abbreviations: F, female; M, male; OFC, occipitofrontal circumference; SDS, standard deviation scores; N/P, not performed; N/A, no longer available; N/K, not known; (✓), indicates presence of a feature in an affected subject; IQ, Intelligence quotient (Wechsler Adult Intelligence Scale); AS, absence seizures; GTC, Generalised tonic clonic seizures; CT, computerised tomography; [a], original imaging no longer available.Height, weight and OFC Z-scores were calculated using a Microsoft Excel add-in to access growth references based on the LMS method[1] using a reference European population[2]

Supplementary References

1. Pan H, Cole TJ. LMS growth, a Microsoft Excel add-in to access growth references based on the LMS method. Version 2.77, http://www.healthforallchildren.co.uk/;2012
2. Cole TJ, Freeman JV, Preece MA: British 1990 growth reference centiles for weight, height, body mass index and head circumference fitted by maximum penalized likelihood. Stat Med 1998, 17(4):407–429
